# Quality of Life Analysis of Patients Treated with Percutaneous Hepatic Perfusion for Uveal Melanoma Liver Metastases

**DOI:** 10.1007/s00270-024-03713-0

**Published:** 2024-04-08

**Authors:** T. M. L. Tong, M. Fiocco, J. J. van Duijn-de Vreugd, J. Lutjeboer, F. M. Speetjens, F. G. J. Tijl, M. E. Sitsen, R. W. M. Zoethout, C. H. Martini, A. L. Vahrmeijer, R. W. van der Meer, C. S. P. van Rijswijk, A. R. van Erkel, E. Kapiteijn, M. C. Burgmans

**Affiliations:** 1 Interventional Radiology Research (IR2) Group, Department of Radiology, C2-S, Leiden University Medical Center, Albinusdreef 2, 2333 ZA Leiden, The Netherlands; 2 Department of Medical Oncology, Leiden University Medical Center, Leiden, The Netherlands; 3 Mathematical Institute, Leiden University, Leiden, The Netherlands; 4 Medical Statistics Section, Department of Biomedical Data Science, Leiden University Medical Center, Leiden, The Netherlands; 5 Department of Extra Corporal Circulation, Leiden University Medical Center, Leiden, The Netherlands; 6 Department of Anesthesiology, Leiden University Medical Center, Leiden, The Netherlands; 7 Department of Surgery, Leiden University Medical Center, Leiden, The Netherlands

**Keywords:** Uveal melanoma, Liver metastases, Percutaneous hepatic perfusion, Melphalan, Chemotherapy

## Abstract

**Purpose:**

Percutaneous hepatic perfusion with melphalan (M-PHP) is a minimally invasive therapy with proven efficacy in patients with uveal melanoma (UM) liver metastases. M-PHP is associated with a short hospital admission time and limited systemic side effects. In this study, we assessed quality of life (QoL) in UM patients treated with M-PHP.

**Materials and Methods:**

A prospective, single-center study including 24 patients treated with M-PHP for UM metastases to the liver. QoL questionnaires were collected at baseline, on day 2/3 after M-PHP, and on day 7 and day 21 after M-PHP, according to study protocol. The results were scored according to EORTC-QLQ C30 global health status (GHS), functional scales, and symptom scales. The difference in scores at baseline and subsequent time points was analyzed with the Wilcoxon signed-rank test and multiple testing Bonferroni correction. Adverse events (AE) were registered up to 30 days after M-PHP according to CTCAE v5.0.

**Results:**

Twenty-four patients (14 males; median age 63.0 years) completed 96 questionnaires. Most scores on all scales declined on day 2/3 after M-PHP. On day 21 after M-PHP, 12 out of 15 scores returned to baseline, including median GHS scores. Three variables were significantly worse on day 21 compared to baseline: fatigue (6–33; *p* = 0.002), physical functioning (100 vs 86.7; *p* = 0.003), and role functioning (100 vs 66.7; *p* = 0.001). Grade 3/4 AEs consisted mainly of hematological complications, such as leukopenia and thrombopenia.

**Conclusion:**

M-PHP causes fatigue and a decline in physical and role functioning in the 1st weeks after treatment, but GHS returns to baseline levels within 21 days.

**Level of Evidence 3:**

Cohort study.

**Graphical Abstract:**

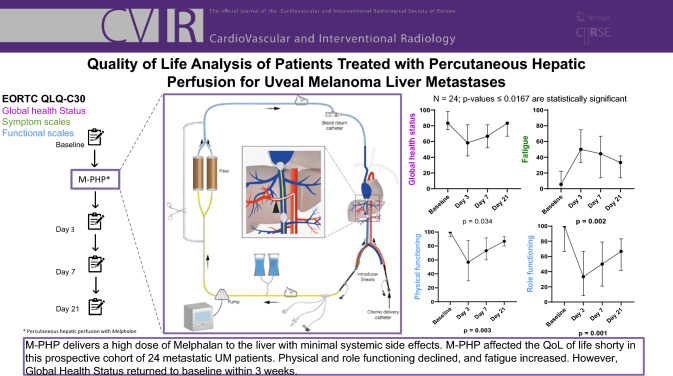

## Introduction

Percutaneous hepatic perfusion with melphalan (M-PHP) is a minimally invasive procedure that allows delivery of a high dose of chemotherapy directly to liver tumors, with limited systemic exposure. In this procedure, the liver is isolated from the systemic circulation with percutaneously inserted catheters, and melphalan is administered through the hepatic artery [1, 2].

M-PHP is a palliative treatment for hepatic metastases from uveal melanoma (UM). Approximately 50% of patients with UM develop metastases throughout the course of the disease, and in up to 90% of these patients, metastases are confined to the liver [3]. Metastatic UM has a dismal prognosis, and limited systemic therapeutic options are available. To date, tebentafusp is the only systemic therapy for which overall survival benefit has been shown in a randomized controlled trial. Treatment with tebentafusp is, however, limited to HLA-A*02:01-positive patients [4].

Mounting evidence has led to the recent approval by the US Food and Drug Administration (FDA) of M-PHP as a treatment for patients with unresectable UM liver metastases. Preliminary results from a recent phase III trial showed a significantly better progression-free survival (PFS) in patients treated with M-PHP compared to best alternative care (BAC) (9.0 months vs 3.1 months) and a median overall survival (OS) of 21 versus 14 months [5]. Superiority of M-PHP over BAC has previously been shown in another randomized trial [6]. The evidence for the efficacy of M-PHP in patients with UM liver metastases thus seems compelling, but median OS after treatment is still less than 2 years [7–11]. Given the limited life expectancy in this patient group, it is of utmost importance to weigh the survival benefit against the toxicity and alterations in quality of life (QoL) after treatment. M-PHP has been proven to be safe with predominantly transient and self-limiting hematological adverse events, but little is known about QoL after M-PHP [2, 12, 13].

There is a lack of prospective studies that have reported on QoL of patients treated with M-PHP [8, 14, 15]. Available data mainly come from studies with retrospective design or risk of bias. As part of a post-market registry study in patients undergoing M-PHP for metastatic UM, we prospectively collected patient-reported outcome measures (PROMs) to assess quality of life.

## Methods

### Study Design

The study was designed as a prospective cohort study. The study protocol was reviewed and approved by the institutional medical ethical board, and informed consent was obtained from all patients. The study was registered on ClinicalTrials.gov with number NCT03266042. The study was terminated early due to slow recruitment as competing clinical trials were on-going in the participating centers. As part of the study, PROMs were collected using QoL questionnaires, and these are reported in this analysis.

### Patient Selection

All consecutive patients undergoing a first M-PHP for hepatic metastases from UM were eligible for participation and asked to participate. Patients were included in this QoL analysis if all questionnaires were filled out.

### Intervention

#### M-PHP Procedure

The procedure has previously been described in detail [1, 14, 16–18]. In short, M-PHP was performed under general anesthesia. Percutaneous vascular access was created to both internal jugular veins, the right common femoral vein, and the femoral artery. Three mg/kg melphalan was administered directly to the hepatic artery (maximum of 220 mg). Access to the femoral artery was closed with a vascular closure device, and the venous access was closed by manual compression. Patients were discharged after 2 days if no complications occurred. Granulocyte colony-stimulating factor (G-CSF) was given within 48 h after M-PHP.

### Outcomes

#### Quality of Life

Patients were requested to fill out the European Organization for Research and Treatment of Cancer (EORTC) QLQ-C30 version 3 questionnaires, a validated questionnaire developed to assess the quality of life of cancer patients [19]. Questionnaires were collected at baseline (before M-PHP), day 2/3 after M-PHP, on day 7, and on day 21, according to study protocol. The EORTC QLQ-C30 consists of questions regarding the global health status (GHS), symptom scales, and functional scales [19]. Symptom scales consist of fatigue, nausea/vomiting, pain, dyspnea, insomnia, appetite loss, constipation, diarrhea, and financial difficulties scores. The functional scales consist of physical, role, emotional, cognitive, and social functioning scores. For the global health status (GHS) and functional scales, a higher score indicates better performance with a maximum score of 100. For the symptom scales, a higher score indicates more symptoms (worse performance), also with a maximum score of 100. For all patients, QoL was measured for the first M-PHP procedure only.

#### Adverse Events

Adverse events (AEs) up to 30 days after M-PHP were assessed according to Common Terminology Criteria for Adverse Events version 5.0 (CTCAEv5.0).

### Statistical Analyses

The Wilcoxon signed-rank test was used to analyze the differences between the scores at baseline and subsequent time points. Bonferroni multiple testing correction was applied when testing difference between timepoints, leading to the adjusted *p*-values of ≤ 0.0167 to be considered statistically significant. Statistical analyses were performed with SPSS version 29.0 (SPSS Inc., Chicago, IL, USA).

## Results

### Study Population

**Fig. 1:**
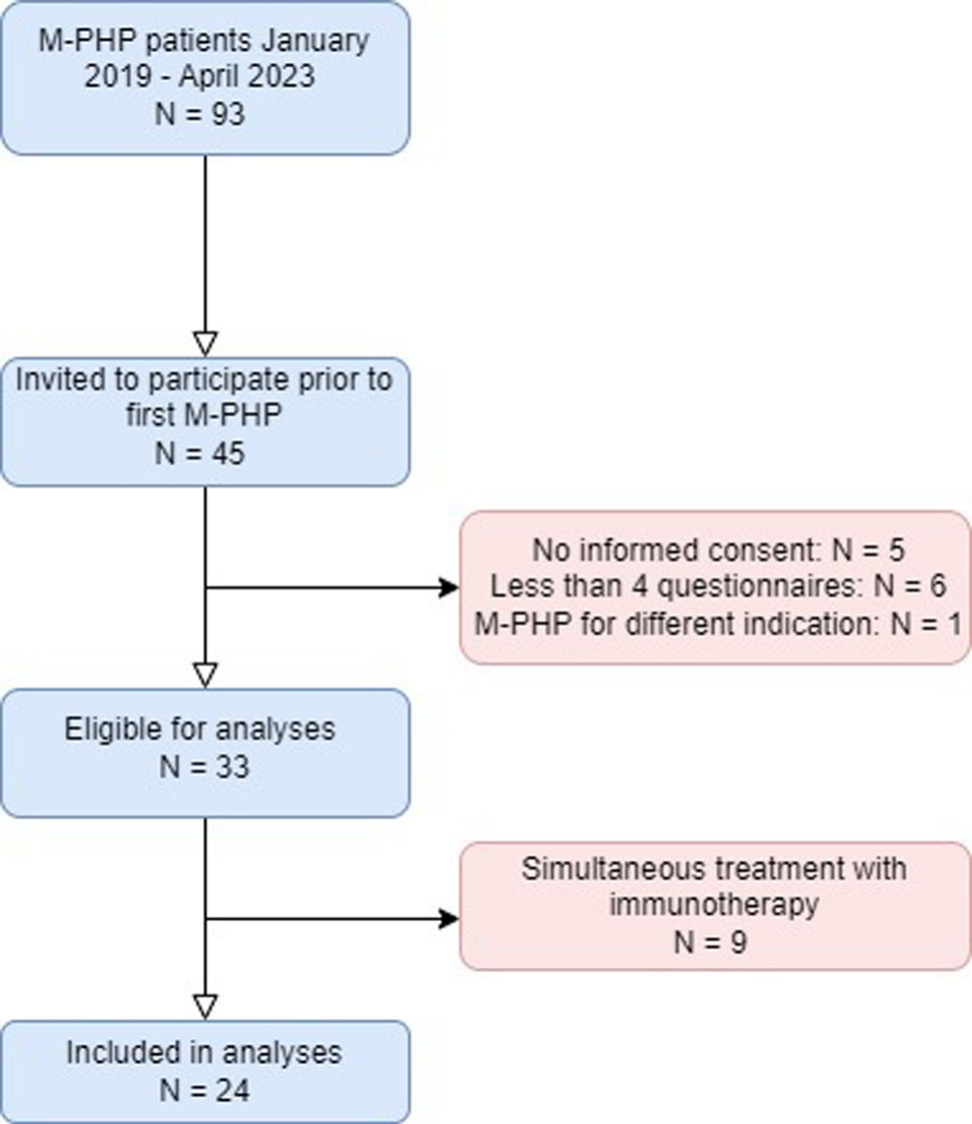
Flowchart of patients. *M-PHP* Percutaneous hepatic perfusion with melphalan

Ninety-three patients were treated with M-PHP between January 2019 and April 2023, from which 45 patients consented to participate prior to the first M-PHP. Thirty-three patients filled in all four questionnaires and were eligible for analyses. From this group, nine patients received combination treatment with M-PHP and immunotherapy as part of a randomized phase II trial. These patients were excluded from the analyses as all study data were under embargo until completion of the trial, resulting in a study cohort of 24 patients (Fig. 1).

The study population consisted of 14 males and 10 females. The median age was 63.0 years (range 47–74). Twelve patients underwent enucleation as treatment for the primary tumor, 10 patients were treated with radiotherapy. Nine patients received prior treatment for hepatic metastases, consisting of ablation (*n* = 5), surgical resection (*n* = 2), or ablation of a liver metastases with cryoablation of a lesion in the sacrum (*n* = 1). One patient received systemic treatment with immunotherapy prior to treatment with M-PHP. All patients presented with metachronous metastases and multifocal disease (Table 1).

**Table 1:** Baseline characteristics

	*N*	%
Number of patients	24	100
Gender		
Male	14	58.3
Female	10	41.7
Age in years [median (range)]	63.0 (47–74)	
Tumor characteristics		
Treatment primary tumor		
Enucleation	12	50
Radiotherapy	10	41.7
Enucleation + radiotherapy	1	4.2
Radiotherapy + endoresection	1	4.2
Prior treatment metastases		
Ablation	5	20.8
Surgical resection	2	8.3
IPI/NIVO*	1	4.2
Ablation liver + cryoablation sacrum	1	4.2
Type of tumor		
Metachronous	24	100
Synchronous	0	0
Multifocal disease		
Yes	24	100

*IPI* ipilimumab, *M-PHP* percutaneous hepatic perfusion with melphalan, and *NIVO* nivolumab

*IPI/NIVO prior to M-PHP, unrelated to the randomized phase II trial

### Quality of Life Scores

**Fig. 2:**
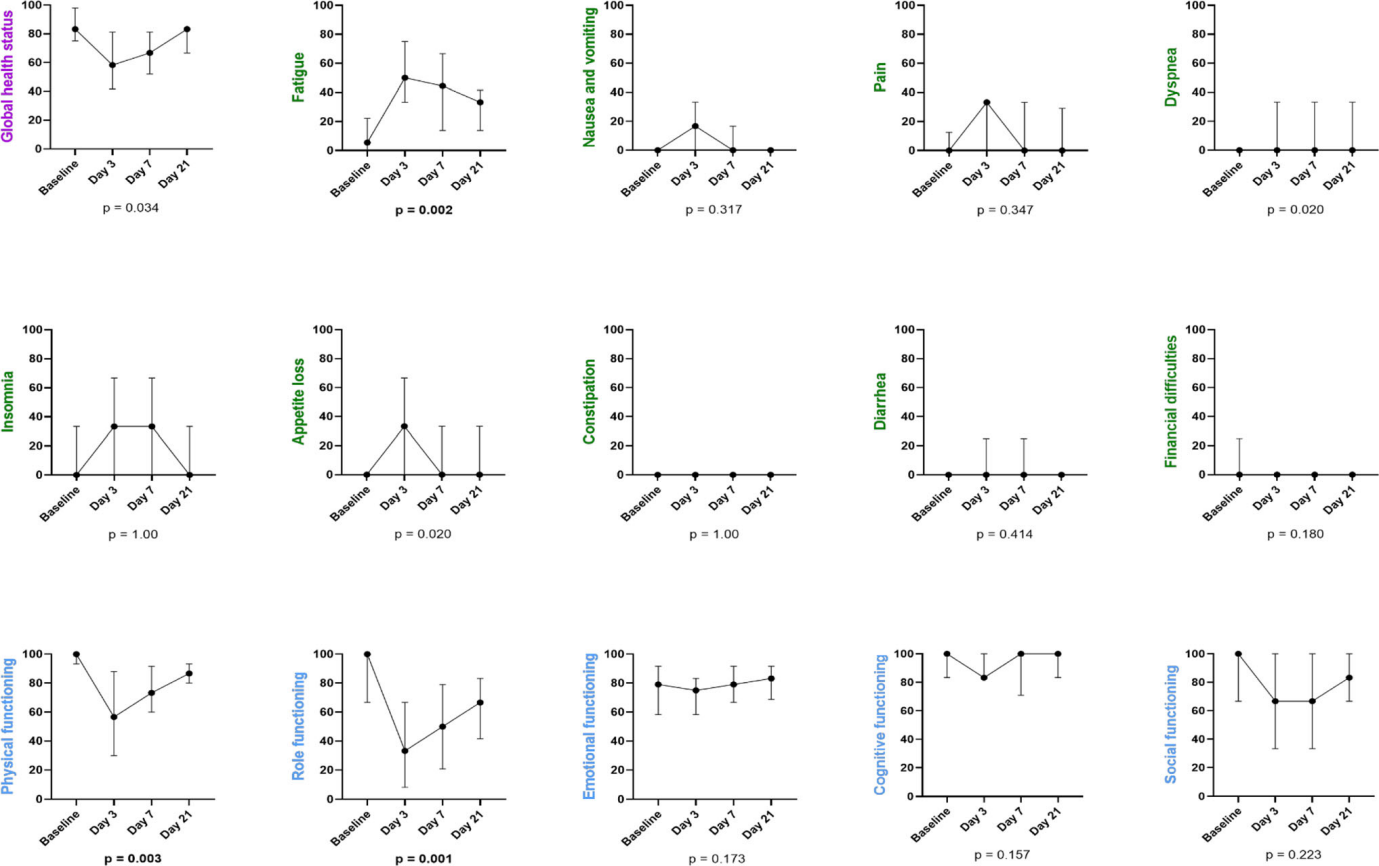
Scale scores EORTC QLQ-C30 as median with interquartile ranges. For global health status scale and functional scales, a higher score indicates better quality of life or functional status. For the symptom scales, a higher score indicates more symptoms. P-values were based on the comparison between score at baseline and on day 21

A total of 96 questionnaires were analyzed of the 24 patients. The scores per scale (global health status, symptom scales, and functional scales) are presented in Table 2 and Fig. 2.

**Table 2:** Scores per scale according to EORTC QLQ-C30 questionnaire

Scale	Scores [median (min–max)]						
	Baseline (*N* = 24)	Day 2 or 3 after M-PHP (*N* = 24)	*p*-value	Day 7 after M-PHP (*N* = 24)	*p*-value	Day 21 after discharge (*N* = 24)	*p*-value (0.0167)
*Global health status/QoL*							
Global health status (QL2) score	83 (50–100)	58 (17–100)	< 0.001	67 (17–100)	< 0.001	83 (33–100)	0.034
*Symptom scales/items*							
Fatigue (FA) score	6 (0–67)	50 (0–100)	< 0.001	44 (0–89)	< 0.001	33 (0–100)	**0.002**
Nausea and vomiting (NV) score	0 (0–17)	17 (0–100)	< 0.001	0 (0–83)	0.014	0 (0–83)	0.317
Pain (PA) score	0 (0–67)	33 (0–100)	0.005	8 (0–67)	0.056	0 (0–83)	0.347
Dyspnea (DY) score	0 (0–33)	0 (0–67)	0.023	0 (0–67)	0.009	0 (0–67)	0.020
Insomnia (SL) score	0 (0–67)	33 (0–100)	0.001	33 (0–100)	0.010	0 (0–67)	1.00
Appetite loss (AP) score	0 (0–33)	33 (0–100)	< 0.001	0 (0–100)	0.004	0 (0–100)	0.020
Constipation (CO) score	0 (0–33)	0 (0–100)	0.034	0 (0–100)	0.317	0 (0–33)	1.00
Diarrhea (DI) score	0 (0–33)	0 (0–67)	0.059	0 (0–100)	0.131	0 (0–100)	0.414
Financial difficulties (FI) score	0 (0–67)	0 (0–33)	0.102	0 (0–67)	0.317	0 (0–33)	0.180
*Functional scales/items*							
Physical functioning (PF2) score	100 (60–100)	57^a^ (7–100)	< 0.001	73 (40–100)	< 0.001	87 (60–100)	**0.003**
Role functioning (RF2) score	100 (50–100)	33* (0–100)	< 0.001	50 (0–100)	< 0.001	67 (17–100)	**0.001**
Emotional functioning (EF) score	79 (0–100)	75 (25–100)	0.387	79 (25–100)	0.774	83 (25–100)	0.173
Cognitive functioning (CF) score	100 (67–100)	83 (67–100)	0.001	100 (67–100)	0.046	100 (67–100)	0.157
Social functioning (SF) score	100 (33–100)	67** (0–100)	< 0.001	67 (0–100)	0.007	83** (0–100)	0.223

For global health status scale and functional scales, a higher score indicates better quality of life or functional status. For the symptom scales, higher score indicates more symptoms

Bold values indicate the significant results

*M-PHP* percutaneous hepatic perfusion with melphalan and *QoL* quality of life

^a^based on 22 observations

*based on 21 observations

**based on 23 observations

The median GHS score was 83 at baseline. Compared to baseline, on day 2/3 post M-PHP, the median score decreased to 58 (*p* < 0.001). Similarly, the median score on day 7 after M-PHP was significantly lower compared to baseline (67; *p* < 0.001). On day 21, the score restored to baseline value 83 (*p* = 0.034). One patient had a markedly lower score compared to the rest of the group: a decrease in absolute GHS score of 50 points from 83 at baseline to 33 on day 21. The deterioration in GHS score could be attributed to a grade 3 post-procedural hemorrhage in the groin, for which transfusion of two units of red blood cells followed.

All symptom scores started at median 0, except for the fatigue score (Table 2 and Fig. 2). On day 2/3 after M-PHP, patients gave a median score of 17 for nausea/vomiting (*p* < 0.001) and a median score of 33 for pain (*p* = 0.005), insomnia (*p* = 0.001), and appetite loss (*p* < 0.001). The scores for dyspnea, constipation, diarrhea, and financial difficulties did not show statistically significant change compared to baseline (*p* > 0.0167 for all). The median fatigue score was 6 at baseline and increased to 50 at day 2/3 (*p* < 0.001). On day 7 after treatment, fatigue (44; *p* < 0.001), nausea/vomiting (0; *p* = 0.014), dyspnea (0; *p* = 0.009), insomnia (33; *p* = 0.010), and appetite loss (0; *p* = 0.004) were higher compared to baseline. The median scores for pain, constipation, diarrhea, and financial difficulties restored to baseline on day 7 (*p* > 0.0167 for all). On day 21, only fatigue had a higher median score compared to baseline (33; *p* = 0.002), while all other symptom scores had returned to baseline (*p* > 0.0167 for all).

All functional scores started with median score 100, except for the emotional functioning score (Table 2 and Fig. 2). Compared to baseline, physical functioning (57; *p* = 0.001), role functioning (33; *p* < 0.001), cognitive functioning (83; *p* = 0.001), and social functioning (67; *p* < 0.001) showed a significant decline on day 2/3 after M-PHP. The median emotional functioning score was 79 at baseline and 75 on day 2/3 (*p* = 0.387). On day 7 after M-PHP, physical functioning (73; *p* < 0.001), role functioning (50, *p* < 0.001), and social functioning (67; *p* = 0.007) scored significantly lower compared to baseline. Emotional functioning (79; *p* = 0.774) and cognitive functioning (100; *p* = 0.046) restored to baseline values. On day 21, the scores for physical (87; *p* = 0.003) and role functioning (67; *p* = 0.001) remained significantly lower compared to baseline, while the other scores restored to baseline values (*p* > 0.0167 for all).

### M-PHP Procedures

The M-PHP procedures had a median duration of 3.2 h (range 2.3–4.8 h). The median melphalan dose for the whole cohort was 220 mg (range 153–220 mg; Table 3).

**Table 3:** M-PHP procedure characteristics

	*N*	%
Number of M-PHPs	24	100
M-PHP duration in hours [median (range)]	3.2 (2.3–4.8)	
Melphalan dose in mg [median (range)]	220 (153—220)	
Periprocedural complications^+^		
None	22	91.7
Yes	2	8.3
Hypothermia grade 4*	1	4.2
Intraoperative arterial injury grade 1	1	4.2

*M-PHP* percutaneous hepatic perfusion with melphalan

^+^According to Common Terminology Criteria for Adverse Events version 5.0

*Hypothermia combined with hypotension, metabolic acidosis, atrial fibrillation, bradycardia, and ST-depressions, requiring intubation. The patient recovered the same day

### Adverse Events

#### Periprocedural Adverse Events

Two patients experienced periprocedural complications. One patient experienced hypothermia, hypotension, metabolic acidosis, and cardiac complications (atrial fibrillation, bradycardia, and ST-depressions) and needed to stay intubated after the procedure. The patient recovered within hours after the procedure and could be discharged on day 3 without any sequelae. The other patient had a pseudoaneurysm of the common femoral artery treated with thrombin injection (Table 3).

#### Post-procedural Adverse Events

All AEs within 30 days after M-PHP were registered. All patients experienced post-procedural AEs, resulting in a total of 134 reported AEs. Twenty-one (15.7%) of all post-procedural AEs were grade 3 or 4 (Table 4). Nine patients experienced the aforementioned total of 21 grade 3 or 4 AEs, of which 16 were hematological AEs. The median GHS score of these patients was 67 on day 21. This score was not significantly different compared to patients who experienced low-grade AEs (median GHS score 83, *p* = 0.174). Asymptomatic grade 1 or 2 anemia (*n* = 16), thrombopenia (*n* = 13), and increased lactate dehydrogenase (LDH), alanine aminotransferase (ALT), and gamma-glutamyl transferase (GGT) (*n* = 11) were the most frequent AEs. Patients also reported transient nausea (*n* = 6), fatigue (*n* = 5), and alopecia (*n* = 5). Grade 3 or 4 leukopenia and thrombocytopenia were most frequently reported (*n* = 6 each). No deaths occurred during treatment period.

**Table 4:** Post-procedural adverse events within 30 days, according to CTCAE v 5.0

Complication	Grade 1/2 (*N*)	Grade 3 (*N*)	Grade 4 (*N*)
*Hematological*			
Anemia	16	1	
Leukopenia	2	2	4
Thrombopenia	13	1	5
Neutropenia		2	1
*Hepatic*			
Increased LDH	11		
Increased ALT	11		
Increased AST	8	1	
Increased ALP	5		
Increased GGT	11	2	
*Gastrointestinal*			
Nausea		6	
Vomiting	4		
Abdominal pain	1		
*Vascular*			
Post-procedural hemorrhage groin	3	1	
Superficial thrombophlebitis	1		
Hematoma	1		
*Cardiac disorders*			
Atrial fibrillation	1		
Increased troponin^a^	1		
*Infections*			
Febrile neutropenia		1	
Herpes simplex reactivation	1		
*Musculoskeletal and connective tissue*			
Pain extremities	1		
*General*			
Fatigue		5	
Headache	2		
Alopecia	5		
Hypotension^b^	1		
Eye infection		1	
Weight loss	1		
Flu-like symptoms	1		

*LDH* lactate dehydrogenase; *ALT* alanine aminotransferase; *AST* aspartate aminotransferase; *ALP* alkaline phosphatase; *GGT* gamma-glutamyl transferase; and *CTCAE v 5.0* Common Terminology Criteria for Adverse Events version 5.0

^a^Transient increase in the patient with grade 4 hypothermia

^b^Hypotension for 2 days post-procedurally, recovered before discharge

## Discussion

In this prospective study, we showed that M-PHP has limited impact on QoL and is well tolerated in patients treated for metastasized UM. Compared to baseline, the median GHS score showed an initial decline at day 3 after M-PHP but had returned to baseline after 3 weeks (median 83 vs 83 at baseline; *p* = 0.034). Fatigue was the only item of the symptom scale that stayed significantly worse after 3 weeks compared to baseline (median 33 vs 6 at baseline; *p* = 0.002). Regarding the functional scales, physical and role functioning were significantly lower compared to baseline after 3 weeks.

Our results are consistent with the previous studies on QoL after M-PHP. In a prospective study including 35 UM patients treated with 2 cycles of M-PHP, QoL was assessed as a secondary endpoint using EORTC QLQ-C30 v3.0 questionnaires at baseline, 6 weeks after the first M-PHP and second M-PHP, and 6 months after the first M-PHP. In this study, the scores did not significantly differ from baseline, except for physical functioning which was significantly lower 6 weeks after the second M-PHP (*p* = 0.011) [8]. Physical functioning score was restored to normal 3 months later. This study was limited by a low response rate. Completed forms were returned by 51%, 74%, 59%, and 49% of patients at baseline, 6 weeks after first M-PHP, 6 weeks after second M-PHP, and 6 months, respectively. A retrospective analysis including 18 UM patients also reported QoL after M-PHP [14]. In this analysis, QoL was assessed 6 weeks after treatment using a short survey with questions derived from the EORTC QLQ-C30 questionnaire. There were no questionnaires taken prior to M-PHP. Patients were asked to rate their overall health and quality of life after treatment as compared to prior to treatment on a four-point scale. An increase was reported in mean overall health score from 2.3 to 3.3 and mean QoL score from 2.3 to 3.6. Patient satisfaction with M-PHP was rated at a mean of 3.8, indicating that M-PHP was well tolerable.

The decreased physical and role functioning score and increase in fatigue scores during the observation period in our study are possibly related to the reported AEs within 30 days post-treatment. M-PHP allows delivery of a high dose of melphalan with limited systemic exposure. Nevertheless, some systemic exposure to melphalan is not uncommon, and hematological complications, such as thrombocytopenia, leukopenia, and neutropenia, have been reported in up to three-quarters of patients after M-PHP [13]. The degree of systemic exposure to melphalan varies per patient and is related to the filtration rate of the hemofiltration cartridges. In a previous study, pharmacokinetic analyses on blood samples of M-PHP patients were conducted and measured a mean overall filter efficiency of 86% (range 71.1–95.5%) using the same system that was used in our current study (GEN2 Hemofiltration system) [2, 16]. When hematological complications occur, these are usually low grade, and these parameters return to baseline levels within 3 weeks [2]. In our study, we observed 16 grade 3 or 4 hematological AEs in seven patients. Hematological AEs could possibly lead to symptoms of fatigue and subsequent decrease in physical and role functioning. However, tiredness could also be related to the M-PHP procedure itself and the related cardiac strain, or the use of general anesthesia. In our study, all hematological AEs were resolved.

M-PHP was developed as an alternative to isolated hepatic perfusion (IHP), its surgical counterpart [20]. In a randomized multicenter phase III trial (SCANDIUM), 87 patients with isolated UM liver metastases were assigned to IHP or BAC. Median PFS was 7.4 versus 3.3 months (*p* < 0.0001), and median hepatic PFS was 9.1 months versus 3.3 months (*p* < 0.0001), both in favor of the IHP arm [21]. Despite the high efficacy of IHP, it is associated with higher complication and mortality rates and longer procedure times compared to M-PHP, as was described in a recent meta-analysis by Bethlehem et al. [22]. According to their analysis, a higher percentage of complications were reported in IHP studies (39.1%) compared to M-PHP studies (23.8%). Similarly, the 30-day mortality rate was higher for IHP (5.5%) compared to M-PHP (1.8%) [22]. Furthermore, IHP is not repeatable, preventing further treatments in case of disease recurrence. Although no studies have been conducted that compare QoL between IHP and M-PHP, it seems self-evident that QoL after a minimally invasive procedure such as M-PHP would be better as compared to IHP, a major surgical procedure. Limited data are available on QoL of patients with UM metastases treated with other minimally invasive liver-directed therapies. Short-term QoL results after treatment with transarterial radioembolization (TARE) showed stable GHS between baseline and follow-up, albeit for different tumor types [23].

Immunotherapy is another alternative for treating metastatic UM. However, there are notable differences in the target population, toxicity profile, and treatment scheme of systemic therapies. Tebentafusp, an immune-mobilizing monoclonal T-cell receptor, is a treatment that is only applicable for HLA-A*02:01-positive patients. Treatment can lead to AEs such as cytokine release syndrome, rashes, and pyrexia within the first 4 weeks of treatment [4]. Immune checkpoint inhibitors (ICI) can lead to immune-related AEs such as fatigue, rash, nausea or even colitis, inflammation pneumonitis, and interstitial nephritis [24]. The chance is higher when they are applied as combination therapy (anti-CTLA-4 with anti-PD1). Lastly, treatment duration of systemic therapies differs markedly from treatment with M-PHP. ICI treatment consists of multiple infusions over a course of months, depending on the study regimen and/or treatment response. Tebentafusp treatment is weekly, until disease progression. Treatment course with M-PHP is usually considerably shorter. However, there are currently no prospective studies that have compared QoL after M-PHP with systemic therapy.

This study is not without limitations. Firstly, it is a single-center study in a small number of patients. The findings may be subject to survey bias as only a small percentage of patients undergoing M-PHP during the study period were willing to participate and returned all questionnaires. Secondly, QoL was only evaluated up until 3 weeks after the first M-PHP, before the second M-PHP was performed. However, this is one of the few prospective studies investigating the QoL of patients being treated with M-PHP.

## Conclusion

Our study shows that M-PHP has limited impact on QoL of patients with metastasized UM. Despite moderate decline in fatigue and physical and role functioning scores, the general GHS returns to baseline within 3 weeks.
