# Correction to: Quality of Life Analysis of Patients Treated with Percutaneous Hepatic Perfusion for Uveal Melanoma Liver Metastases

**DOI:** 10.1007/s00270-024-03733-w

**Published:** 2024-05-06

**Authors:** T. M. L. Tong, M. Fiocco, J. J. van Duijn-de Vreugd, J. Lutjeboer, F. M. Speetjens, F. G. J. Tijl, M. E. Sitsen, R. W. M. Zoethout, C. H. Martini, A. L. Vahrmeijer, R. W. van der Meer, C. S. P. van Rijswijk, A. R. van Erkel, E. Kapiteijn, M. C. Burgmans

**Affiliations:** 1 Interventional Radiology Research (IR2) Group, Department of Radiology, C2-S, Leiden University Medical Center, Albinusdreef 2, 2333 ZA Leiden, The Netherlands; 2 Mathematical Institute, Leiden University, Leiden, The Netherlands; 3 Medical Statistics Section, Department of Biomedical Data Science, Leiden University Medical Center, Leiden, The Netherlands; 4 Department of Medical Oncology, Leiden University Medical Center, Leiden, The Netherlands; 5 Department of Extra Corporal Circulation, Leiden University Medical Center, Leiden, The Netherlands; 6 Department of Anesthesiology, Leiden University Medical Center, Leiden, The Netherlands; 7 Department of Surgery, Leiden University Medical Center, Leiden, The Netherlands

**Correction to: Cardiovasc Intervent Radiol** 10.1007/s00270-024-03713-0

In the original article, the second affiliation of the corresponding author Thaïs Tong is incorrect:

Mathematical Institute, Leiden University, Leiden, The Netherlands.

The correct second affiliation of the corresponding author Thaïs Tong is:

Department of Medical Oncology, Leiden University Medical Center, Leiden, The Netherlands.

The original article has been corrected.

